# Giant Appendicular Mucocele Due to Mucinous Cystadenoma

**DOI:** 10.5005/jp-journals-10018-1197

**Published:** 2016-12-01

**Authors:** Mehmet Sertkaya, Arif Emre, Eyüp Mehmet Pircanoglu, Onur Peker, Emrah Cengiz, Mustafa Karaagaç

**Affiliations:** 1 Department of General Surgery, Kahramanmaraş Sütçü İmam Üniversitesi, Kahramanmaraş, Turkey; 2 Department of General Surgery, Necip Fazil State Hospital, Kahramanmaraş, Turkey; 3 Department of Pathology, Kahramanmaraş Sütçü İmam Üniversitesi Kahramanmaraş, Turkey

**Keywords:** Appendectomy, Appendicitis, Cystadenoma, Giant mucocel.

## Abstract

Mucocele of the appendix is a rare clinicopathological entity simulating acute appendicitis. The most common form of the mucocele is cystadenoma, which is characterized by luminal dilatation producing large amounts of mucin. We present a new case of a giant mucocele of appendix with mucinous cystadenoma. A 61-year-old female was admitted with complaints of severe lower right quadrant pain. Ultrasonography and computed tomography (CT) suggested that it was a mucocele, but due to severity of pain, she underwent an emergency operation. Fortunately, without a perforation, it was a giant mucocele and the operation was terminated with an uneventful appendectomy with segmental cecal resection. The histopathological evaluation of the specimen reported to be a mucocele with mucinous cystadenoma with negative surgical margins. The patient was discharged postoperative 6th day, and a control colonoscopy and abdominal CT was planned for 6 months following surgery. Appendicular mucocele is rare and difficult to diagnose preoperatively, and sometimes it may be of large size which increases the risk of perforation. Pseudomyxoma peritonei (PP) is the most feared complication of mucocele perforation. Appendectomy with negative margins is a requirement for adequate treatment for most cases. Utmost care should be taken during surgery to avoid perforation of mucocele.

**How to cite this article:**

Sertkaya M, Emre A, Pircanoglu EM, Peker O, Cengiz E, Karaagaç M. Giant Appendicular Mucocele Due to Mucinous Cystadenoma. Euroasian J Hepato-Gastroenterol 2016;6(2):186-189.

## INTRODUCTION

Appendicular mucocele is a rare pathological entity characterized by cystic dilatation of the appendix due to abnormal appendiceal mucinous secretion by benign or malign lesions. Retention cyst or simple mucocele, mucosal hyperplasia, cystadenoma, and cystadenocarcinoma are four main groups of mucoceles. Cystadenoma is the most common form and is characterized by tubular adenomatous epithelium with varying degree of epithelial atypia. It manifests itself in different clinical features, such as producing large amounts of mucin which could sometimes result in giant luminal dilatation that might perforate spontaneously or during surgery. Perforation of a mucocele can cause pseudomyxoma peritonei (PP) which is the most feared complication leading to a worse clinical condition. The most important point is preoperative accurate diagnosis and maximum care about perforation risk during surgery to avoid having to deal with PP. The most important diagnostic tools are ultrasonography and computed tomography (CT). Periodic follow-up after surgery should be emphasized due to possible recurrences. Here, we present a new case of a giant appendicular mucocele with mucinous cystadenoma.

## CASE REPORT

**Fig. 1:**
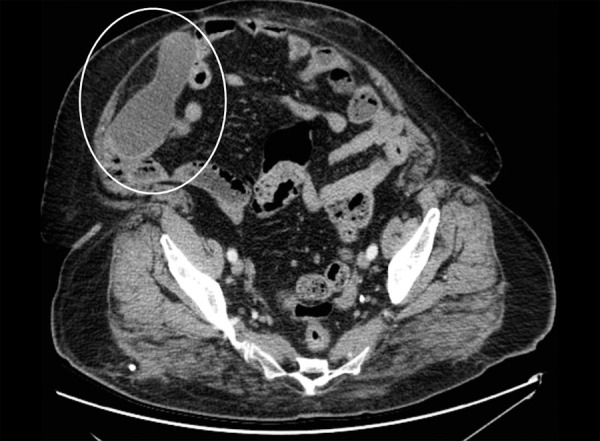
Cystic mass

**Fig. 2:**
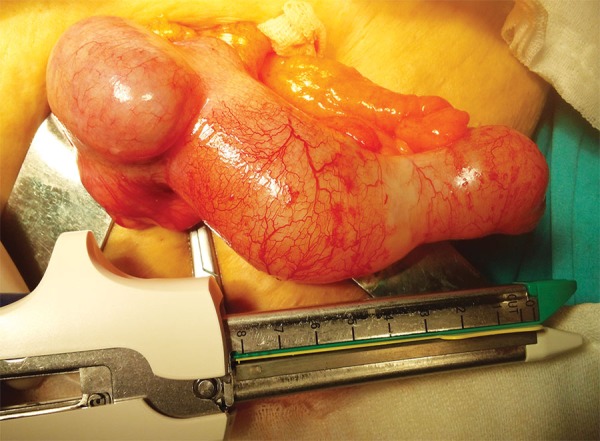
A mucocele

**Fig. 3:**
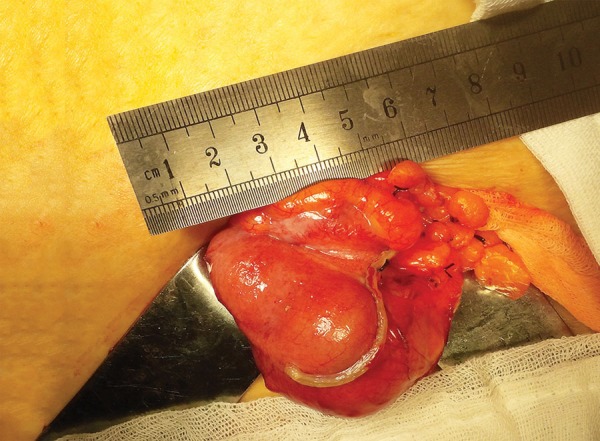
Surgical intervention

**Fig. 4:**
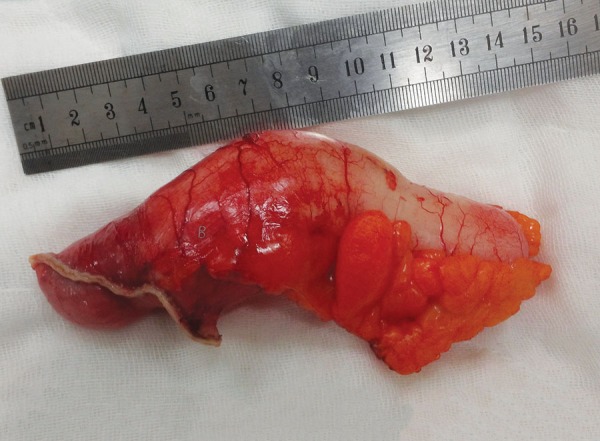
Surgical intervention

**Fig. 5:**
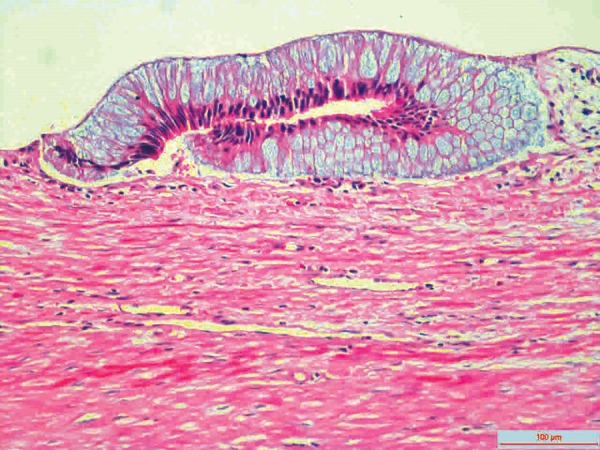
Histopathological features of the lesion

A 61-year-old female patient with past history of hypertension, diabetes mellitus, hyperlipidemia, and a coronary artery bypass surgery 8 years ago was admitted to the emergency department with complaints of sudden onset of a severe blunt pain in the right lower quadrant. She had no episodes of vomiting or nausea, but had a chronic constipation complaint. On her general and physical examination, she was moderately obese, had right lower quadrant tenderness, and guarding and rebound was positive. The blood tests revealed leukocytosis around 13.5 K/µL, an elevated C-reactive protein value around 124 mg/L, and an elevated glucose value around 340 mg/dL. Other biochemical values were at within normal limits. The abdominal ultrasonography detected a 113 × 44 × 55 mm-sized, hypoanechoic encapsulated lesion with distinct contours located in the right lower quadrant adjacent to anterior wall of the abdomen. A planned abdominal CT to evaluate the differential diagnosis and the presence of additional pathologies revealed a tubular-shaped cystic mass without any solid components, superiorly extending to anterior abdominal wall from the level of right ileocecal valve, about 14 × 5 cm in size, with a thin wall, having a density of about 20 HU (Fig. 1). Due to patient’s discomfort, assuming a perforation of appendicitis or the cystic mass, she underwent an emergency operation in which McBurney’s incision was performed where an enlarged unperforated appendix was observed, which was clearly a mucocele (Fig. 2). Considering the risk of perforation and consequences, we resected the base of appendix with linear stapler involving some of the cecum for robust surgical margins (Figs 3 and 4). Histopathological examination of the specimen showed a cystically dilated appendix measuring 14 × 5 × 4 cm in size. The luminal content of the cyst measuring 9 × 4.5 cm in size consisted of mucinous fluid and was green fecaloid in the appendiceal lumen. Microscopically, the specimen had features of mucinous cystadenoma placed in the diagnosis (Fig. 5). After monitoring in intensive care and service 2 and 3 days respectively, the patient was discharged, and control colonoscopy and abdominal CT was planned after 6 months following the discharge.

## DISCUSSION

Being a morphological term that describes the transformation of the appendix into a bag full of mucus depending on several etiologies,^1^ the term “mucosal” was first coined by Karl Freiherr von Rokitansky in 1842.^1-3^ It is an obstructive and cystic dilatation of the appendiceal lumen caused by intraluminal accumulation of mucoid material.^2,3^ Although recognized as a pathological condition by Rokitansky, it is claimed that the term “appendicular mucocele” was first defined and used by Feren in 1876.^3,4^ As the name suggests, it is a simple and understandable term which provides ease for surgeons and radiologists to report it before any exact histopathological diagnosis.^1^ The importance of easy recognition of mucocele is to plan an optimum surgical procedure and to avoid rupture as it could result in a worse entity called PP.^1-3^ Being with high mortality rate,^4^ this is the most feared complication of appendiceal mucocele which occurs due to perforation of mucocele by increased intraluminal pressure, spontaneously or during surgical manupulations.^1,3,5^ The term “PP” was first introduced by Werth in 1884^1^ and signifies the presence of pools of mucin- and mucin-secreting cells within the peritoneal cavity. Other complications of mucocele are reported to be intestinal obstruction by intussusception or volvulus, gastointestinal bleeding, ureteral obstruction, and hematuria.^3,5^

Despite its high risk of perforation and serious complications, due to its low incidence rate, mucocele can be difficult to diagnose preoperatively, and thus it is more likely to be diagnosed during surgery or after pathological evaluation.^1-7^ It is reported that the rate of a correct preoperative diagnosis had been achieved in up to 30% of cases according to the published series.^2^ The incidence of mucocele is ranging from 0.07 to 0.7% of all appendectomy specimens,^1-8^ and it affects both sexes between 4th and 7th decades of life,^1,2,4-6^ with a female predominancy.^4-7^ However, there is still disagreement about gender distribution, and different studies have reported different results.^2,3^

Mucocele symptoms are nonspecific^3^ and often mimics acute appendicitis since the most common presenting symptom is right lower quadrant pain.^1^ Other symptoms of mucoceles encountered in symptomatic patients are a history of painless or painful mass sensation in the lower right quadrant, nausea and vomiting due to intestinal obstruction or intussusception, genitourinary complaints, anemia, weight loss, chronic intermittent colic-type pain, abdominal discomfort, and nonspecific complaints like change in bowel habits.^1-5^ Interestingly, almost 50% of cases are asymptomatic and usually diagnosed incidentally during surgery performed for other reasons, or during radiographic imaging.^1-4^ As far as we can understand, becoming symptomatic for mucoceles depends on the size and location of the lesion. In the present case, the diameter of mucocele and appendix together was 14 cm and led to severe lower abdominal pain that justified an emergency operation.

The two most important diagnostic tools for diagnosis are ultrasonography and CT; however, preoperative diagnosis is difficult.^1-5^ Ultrasound may show an encapsulated cystic lesion adjacent to cecum, but it alone may not be sufficient to make the diagnosis.^3^ We can say that CT is a more accurate diagnostic tool and can reveal more clearly a pericecal well-encapsulated cystic formation with thick or thin wall, sometimes accompanied with parietal calcification.^3^ In the present case, ultrasonography and CT suggested that it was a mucocele (Fig. 1), but the patient's discomfort hinted at a peritonitis which led us to take a decision for an urgent operation. There are also benefits noting that we assured there was not any other pathology seen in the abdomen CT, before considering an appendectomy.

Appendiceal mucocele has four subgroups according to the histopathological changes to the underlying epithelium of the appendix: Retention cyst or simple mucocele (20%), appendiceal mucocele with hyperplastic epithelium and moderate luminal dilatation (5–25%), mucocele through mucinous cystadenoma (63–84%), and malignant mucocele through cystadenocarcinoma (11–20%).^1-7^ The most common type among the four groups is mucinous cystadenoma and has an incidence of 0.6% according to the recent series of appendectomy specimens.^8^ Our case also was a mucocele through mucinous cystadenoma.

Cystadenoma is characterized by tubular adenomatous epithelium, with varying degree of epithelial atypia, and morphologically resembles adenomas in the colon.^4,6^ Being a noninvasive tumor, it produces large amounts of mucin with prominent luminal dilatation of up to 6 cm and has an association with perforation risk about 20%.^4,6^ The luminal diameter of the lesion we measured was about 5 cm, and it was about to perforate (Figs 2 and 3).

Cystadenoma and cystadenocarcinoma are the two neoplastic groups of appendiceal mucoceles which encompass about 35% of primary neoplasms of the appendix.^4^ The mentioned two lesions may occur *de novo* or from a simple mucocele preexisting in the appendix.^4^ In these two cases, complete and accurate excision of the appendix is considered curative surgery if histopathologically it can be proven that negative margins are assured, and lymph nodes are not invaded when evaluated perioperatively.^3,4^ If lymph nodes are invaded, a cecum resection or right colectomy is required for curative surgery.^3^ Perioperative assessment of the present case suggested that it was a benign lesion, and there was no sign of lymph node invasion (Fig. 2). So we decided to perform an appendectomy and partial cecal resection with a linear cutter stapler provided the lesion was completely resected (Fig. 3). For the evaluation, we have sent the resected lesion to the pathology. As a result of the suffering from press by the cyst contents, columnar mucinous epithelium atrophy was seen in several areas of staining with hematoxylin and eosin (Fig. 5). So it was diagnosed by pathologist as mucinous cystadenoma with negative margins.

Accordingly, it is known in the literature that for all four groups of mucoceles surgery is the required treatment.^1-7^ For the first two groups which are also classified as nonneoplastic lesions, a simple appendectomy is an adequate treatment option. Due to the fact that the final diagnosis will not be possible before pathological evaluation of the removed lesion, all kinds of mucoceles perforation should be avoided during open or laparoscopic surgery due to the possible most feared complication, PP.^1-8^

While postoperative prognosis of patients with benign mucoceles is reported to be excellent with 5-year survival rates of 91 to 100%, with malignant mucoceles, the 5-year survival rate is markedly reduced due to complications of PP.^4,5^

Because the mucocele of the appendix may be simultaneously accompanied by solid organ tumors of other locations and the later risk of PP, it is reasonable to explore thoroughly during operation and to follow-up patients periodically after surgery. In the present case, the patient was discharged planning a control colonoscopy and a whole abdominal CT to be performed 6 months after surgery.

## CONCLUSION

Mucocele of the appendix is a very rare entity, but often confused with acute appendicitis. The most important diagnostic tools are ultrasonography and CT. Preoperative diagnosis is very important to decide an appropriate surgical procedure, to make sure there is no any other pathologies, and to avoid perforation of the mucocele which can cause PP. Simple appendectomy with negative margins is generally the sufficient and required treatment, but depending on the size and location of the mucocele, sometimes cecum resection or right hemicolectomy may be required. It is important to follow-up periodically after surgery due to the risk of possible recurrences.
